# Exploring Visual Outcomes in Late-Presenting Multisuture Craniosynostosis: A Case Report

**DOI:** 10.7759/cureus.75059

**Published:** 2024-12-03

**Authors:** Arumugam Balraj, Harini Ravikumar

**Affiliations:** 1 Neuro Ophthalmology, Aravind Eye Hospital and Post Graduate Institute of Ophthalmology, Puducherry, IND

**Keywords:** compressive optic neuropathy, craniosynostosis, frontal advancement surgery, optic atrophy, oxycephaly, proptosis

## Abstract

A five-year-old female came with a history of frequent rubbing of the right eye and noticed prolonged elevation of her head since birth, informed by her mother. On ocular examination, the best corrected visual acuity shown in the right eye was 1/60, and the left eye was 6/6, with proptosis in both eyes. Fundus examination showed both eyes having pale discs. General assessment of the patient shows a high, peaked forehead and a shortened, pointed head shape suggestive of oxycephaly. Immediate neuroimaging revealed premature closure of skull bones with narrowing of orbit apex leading to bilateral compressive optic neuropathy, which is suggestive of craniosynostosis. Frontal advancement surgery was done for oxycephaly to relieve compression to the optic nerve. Post-surgery vision improved to 6/24 in the right eye. Recent advancements in surgical techniques and a collaborative team-based approach have significantly enhanced the safety and outcomes of this disease.

## Introduction

Craniosynostosis is a congenital condition in which one or more of the sutures in the skull fuse prematurely, restricting skull growth and leading to an abnormal head shape. These sutures are normally flexible joints that allow the skull to expand as the brain grows; however, when they fuse too early, the skull cannot grow in the affected areas, resulting in compensatory growth in other regions. Oxycephaly is a rare form of craniosynostosis that causes the skull to have a cone or tower-shaped appearance. It occurs due to the premature fusion of the coronal and sagittal sutures, which prevents the skull from growing normally in width [1]. This condition is typically identified shortly after birth and is often treated with surgical intervention aimed at reducing pressure on the brain and correcting the abnormal skull shape. The management of craniosynostosis has seen significant advancements in recent years, with innovations in both surgical techniques and supportive care. Traditional open cranial vault remodeling, once the primary approach, has been refined to improve outcomes and reduce recovery time. If left untreated, oxycephaly can lead to developmental delays and blindness [2-5]. We present this case to highlight key aspects of late-presenting multi-suture craniosynostosis and its management.

## Case presentation

**Figure 1:**
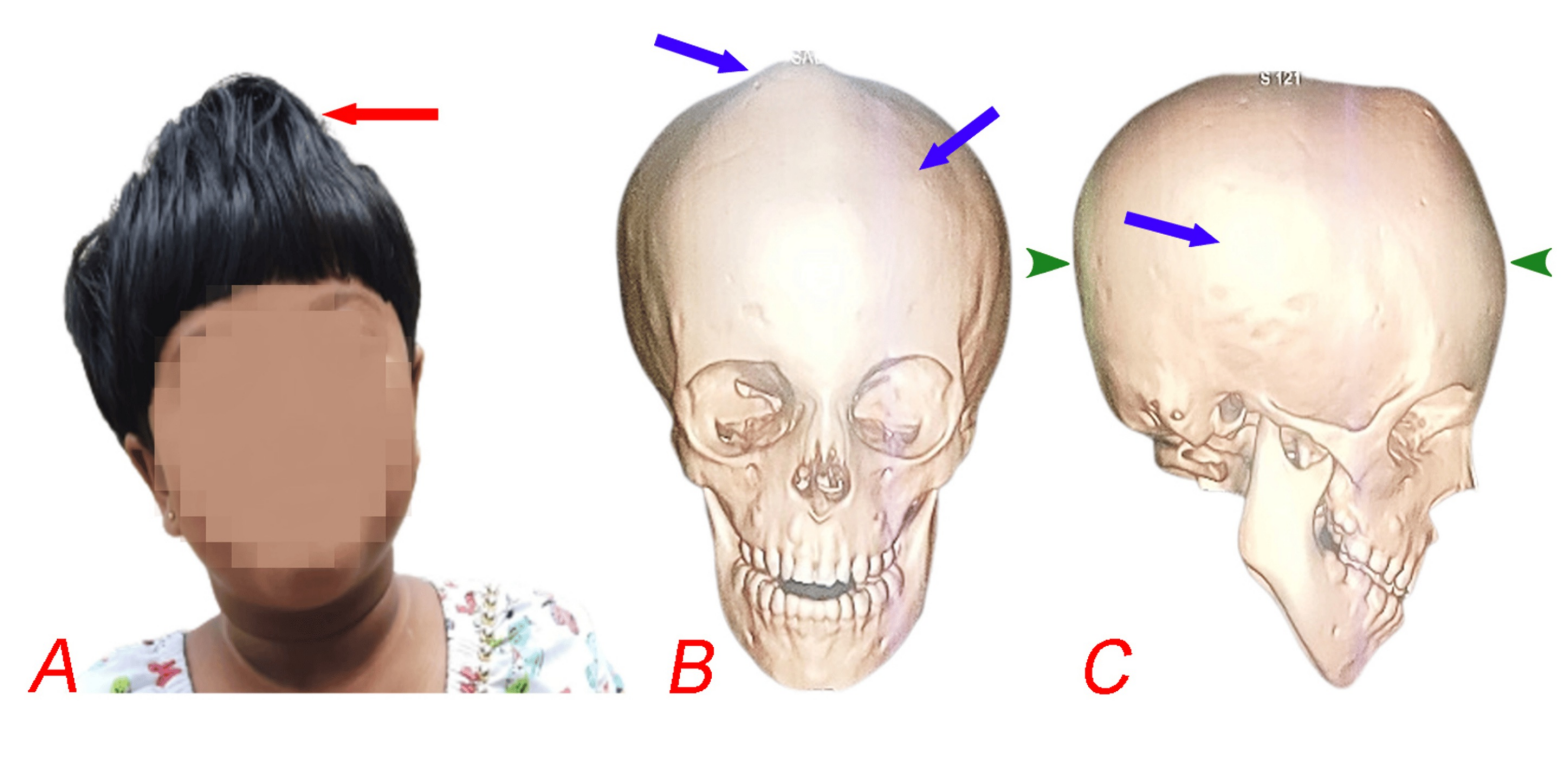
Clinical photograph of the patient (A) showing a high, peaked forehead (red arrow) and a shortened, pointed head shape characteristic of oxycephaly. Frontal view of the 3D CT reconstruction of the skull (B) highlights a significantly elevated and pointed cranial vault, with blue arrows indicating areas of abnormal suture fusion. Lateral view of the 3D CT reconstruction (C) further emphasizes the vertical elongation and foreshortening of the skull (green arrows).

A five-year-old girl, born preterm at 35 weeks via lower-segment cesarean section (LSCS) from a non-consanguineous marriage, had a birth weight of 1.7 kg. She presented with a history of frequently rubbing her right eye. Although her mother did not observe any signs of visual impairment, she had noted an abnormal head shape since birth. The child had no significant systemic history, and her developmental milestones were achieved on time without any delays. General examination revealed alert child with GCS 15. Physical examination revealed a high, peaked forehead and a shortened, pointed head shape indicative of oxycephaly (Figure 1).

**Figure 2:**
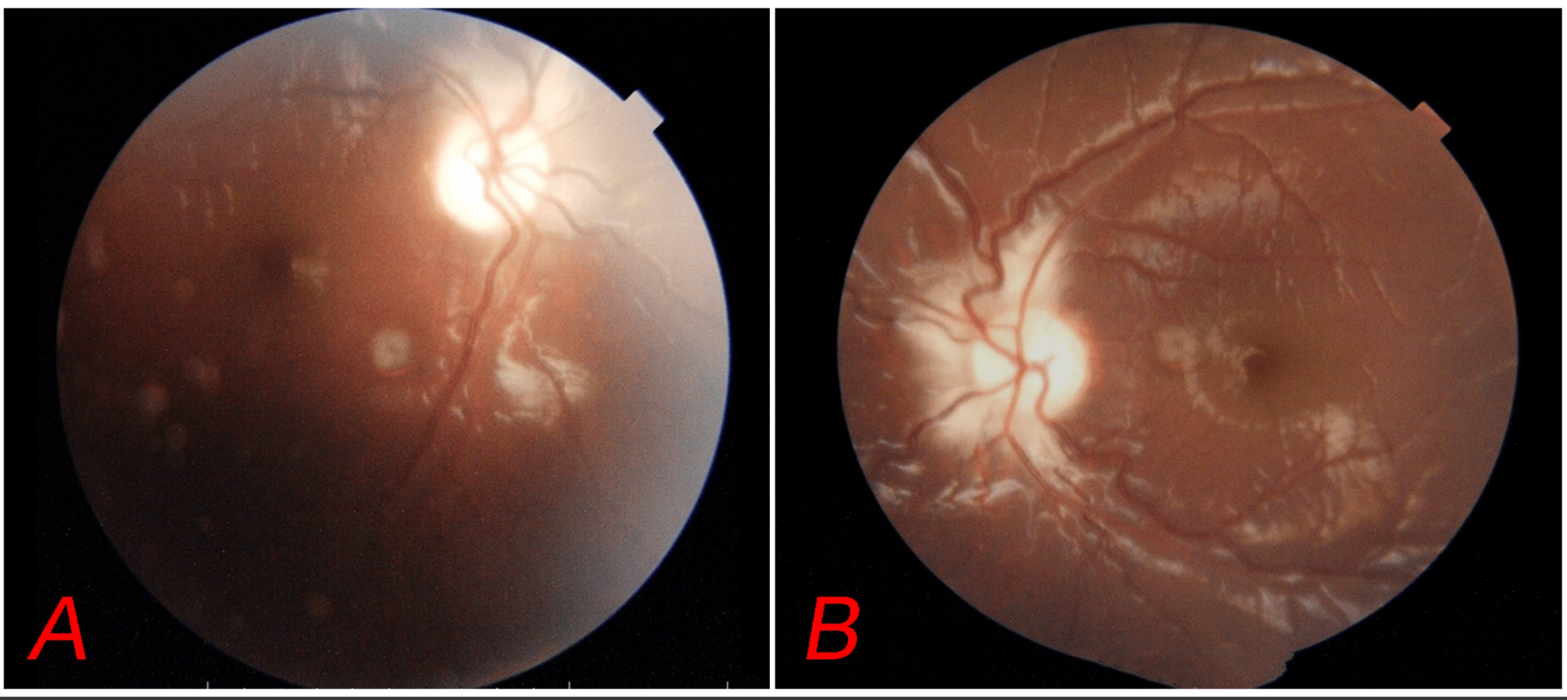
Fundus photograph showing pale disc with peripapillary infarction in both eyes (A and B).

Notable craniofacial features included maxillary recession, a trapezoid-shaped skull with right facial scoliosis, bony nasal septal deviation to the left, and right brow asymmetry. The recorded anthropometric measurements included a head circumference of 44 cm (normal range: 49-51 cm), a weight of 26 kg (normal range: 15-23 kg), a height of 136 cm (normal range: 105-115 cm), and a calculated body mass index (BMI) of 14.1 kg/m² (normal range: 14-17 kg/m²). The ocular assessment revealed a best corrected visual acuity (BCVA) of 1/60 in the right eye and 6/6 in the left eye, with bilateral proptosis. The right eye exhibited defective color vision and generalized field depression. Fundus examination revealed a pale disc with peripapillary infarction in both eyes (Figure 2).

**Figure 3:**
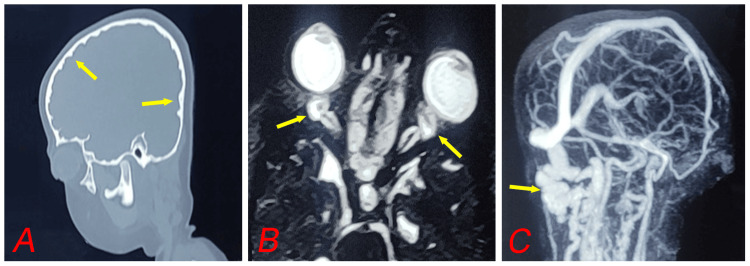
CT brain demonstrating a copper-beaten appearance of the skull, indicative of chronic raised intracranial pressure (ICP) with prominent impressions (arrows) of brain gyri on the inner table of the skull (A), T2-weighted gadolinium-enhanced MRI (CEMRI) of the brain and orbit showing severe optic nerve sheath oedema (arrows) (B), and Magnetic resonance venography (MRV) revealing collateral venous pathways and venous shunting (arrow), indicative of chronic intracranial hypertension and compensatory venous changes (C). CT: computed tomography; CEMRI: contrast enhanced magnetic resonance imaging.

CT brain imaging with 3D reconstruction confirmed pan-sutural craniosynostosis, showing a flattened forehead, prominent pre-coronal hump, and inner calvarial table scalloping, all indicative of raised intracranial pressure (ICP). Contrast-enhanced MRI (CEMRI) with MRV and MRA revealed normal brain architecture but severe optic nerve sheath edema. MRV demonstrated partial transverse sinus hypoplasia with collateral circulation & venous shunts, suggesting long-standing raised ICP (Figure 3).

**Figure 4:**
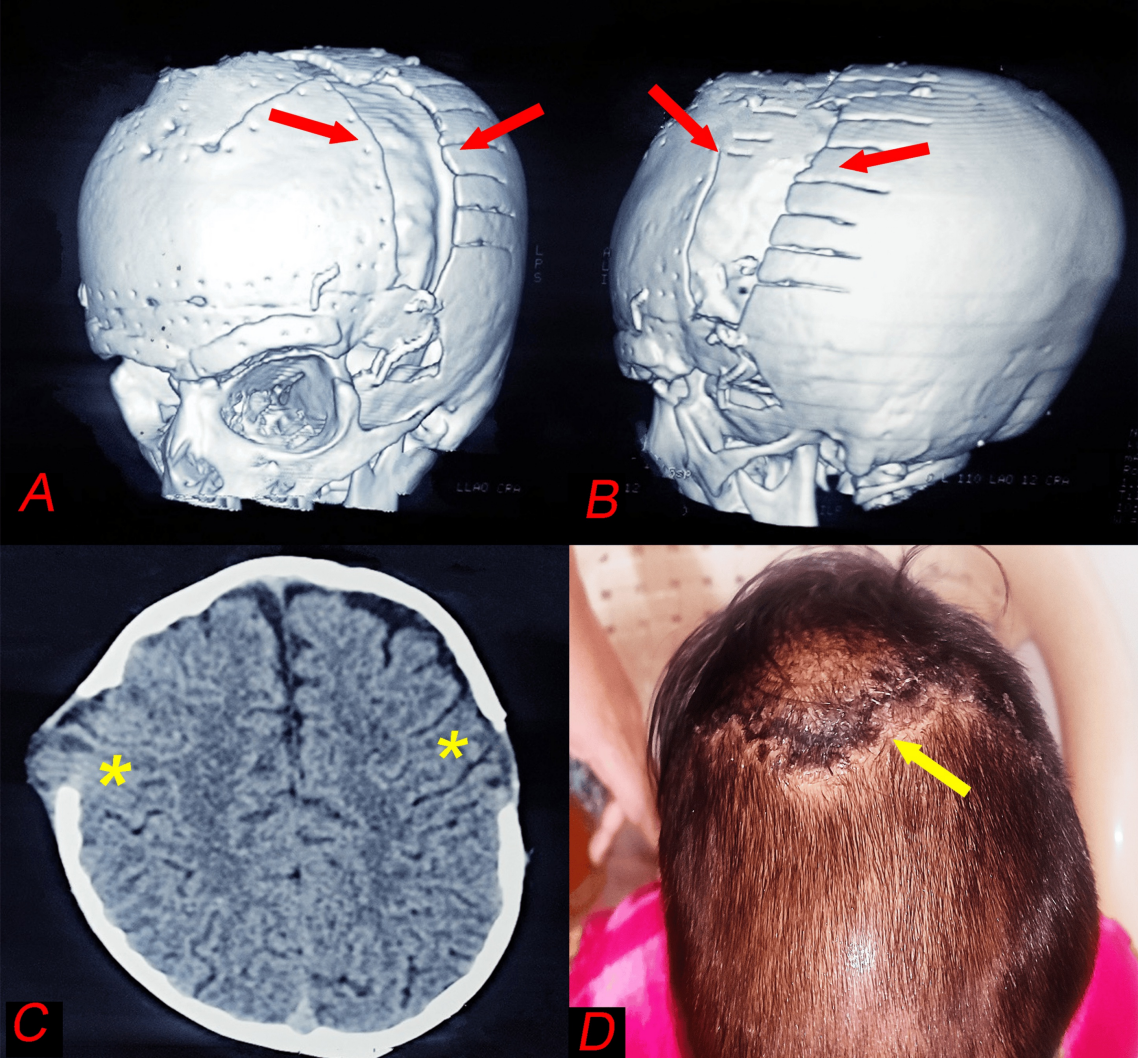
3D reconstructed CT images of the skull following frontal orbital advancement and cranial vault remodeling (red arrows), showing the expanded anterior and posterior cranial vaults with evidence of surgical remodeling and stabilisation (A and B), axial CT image displaying the intracranial view of the cranial vault after surgical intervention, demonstrating craniotomy opening (asterisks) (C), and clinical photograph of the postoperative head showing the healed surgical site with evidence of scalp integrity restoration (yellow arrow) and no significant external deformity (D).

Genetic testing was performed and returned negative. The diagnosis was compressive optic neuropathy due to non-syndromic craniosynostosis (oxycephaly). Treatment involved bilateral frontal-orbital advancement and posterior vault remodeling to relieve optic nerve compression (Figure 4).

**Figure 5:**
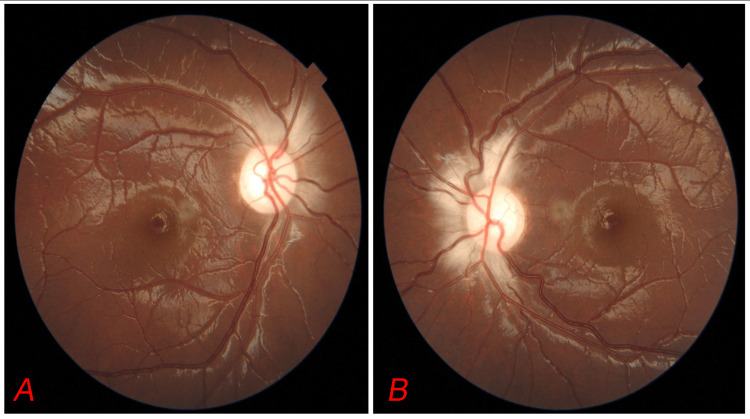
Fundus photographs of both eyes at the one-year follow-up demonstrate a decrease in optic disc pallor with a slight shift toward a pinkish hue, suggesting potential improvement in optic nerve perfusion following surgery (A, B).

Exposure keratitis was identified as an early postoperative complication, with signs observed on the first postoperative day, including mild conjunctival hyperemia, punctate epithelial erosions, and mild lagophthalmos. Management included the use of lubricating eye drops and ointment, along with a temporary tarsorrhaphy, which was removed after one month. These interventions effectively addressed the ocular surface concerns during the early postoperative period. The follow-up schedule for the study included weekly evaluations for the first month, followed by monthly visits for three months, and subsequently monitored every six months till date. Post-surgery, the patient's vision improved to 6/24 in her right eye at the end of one year follow-up (Figure 5).

## Discussion

Craniosynostosis is a relatively rare condition that affects approximately 1 in 1000 infants [1]. Craniosynostosis is a developmental disorder characterized by the premature fusion of cranial sutures, which restricts normal skull and brain growth. Typically, the sutures in an infant’s skull remain open to allow the skull to expand as the brain grows. However, early fusion results in restricted growth perpendicular to the fused sutures (Virchow's law), causing cranial deformities, increased intracranial pressure, and potential neurological deficits [2]. This condition is due to abnormal development of the primitive mesoderm. Recent studies have highlighted a concerning rise in the occurrence of craniosynostoses, a medical condition where the skull sutures fuse prematurely. The increase in craniosynostosis cases is thought to result from both genetic and environmental factors [3]. Environmental risks include fetal head constraint, abnormal fetal position, low amniotic fluid, exposure to teratogens, maternal smoking, and specific medications during pregnancy [3].

About 20% of craniosynostosis cases have a genetic basis, often due to autosomal dominant FGFR (Fibroblast growth factor receptor) mutations-FGFR1 in Pfeiffer's syndrome and FGFR2 in Apert’s and Crouzon’s syndromes [4]. Non-inherited mutations, as observed in our case, account for approximately 50% of cases. Craniosynostosis primarily affects the sagittal suture (60%), followed by the coronal (25%), metopic (15%), and lambdoid sutures (2%) [5]. In our case, the patient presented with premature pansutural craniosynostosis, leading to a tower-shaped skull due to restricted transverse brain growth. The absence of developmental delay, focal neurological deficits, or feeding problems made this condition challenging to detect during infancy. Furthermore, measuring head circumference during routine pediatric vaccination visits can aid in the early detection of craniosynostosis. Regular vision screenings conducted by school teachers play a crucial role in identifying any problems at an early stage and preventing complications like amblyopia. Interdisciplinary team care is important for diagnosis and treatment. Ophthalmic manifestations of craniosynostosis include optic atrophy, exophthalmos, exotropia, nystagmus, and mental deficiency [1]. Optic atrophy is one of the most concerning ocular consequences of craniosynostosis. It occurs due to increased intracranial pressure, mechanical traction, compression, and narrowing of the optic foramen [6]. The occurrence of asymmetric vision loss may stem from disparate sizes of optic canals, as depicted in our case. The primary goals of treatment are to ensure normal brain development and achieve a cosmetically acceptable appearance.

The optimal time for surgery is debated, but it is generally considered between six and twelve months of age [6]. The type and extent of surgery depend on the patient's age and presentation. The options for surgical intervention include open craniotomy and reconstruction or an endoscopic procedure. Endoscopic intervention is more suitable until the age of 6 months when cranial bones are still flexible. After surgery, additional corrections with helmets may be needed for four to six months. After six months, open surgery is preferred due to bone stiffness as done in our case. In particular, frontal advancement open surgery is an effective surgical method for expanding the cranial vault, regardless of age [6,7]. This procedure involves removing the fused sutures and reshaping the forehead, which can significantly reduce the pressure on the optic nerve. Ideally, surgery should be performed in the first year of life [8]. In craniosynostosis surgery, specifically frontal orbital advancement combined with posterior vault remodeling, several factors regarding its benefits and potential complications must be considered. This combined approach addresses both anterior and posterior cranial vaults, facilitating balanced expansion to accommodate normal brain growth while significantly improving craniofacial aesthetics by correcting deformities and enhancing facial symmetry. Additionally, it reduces the risk of elevated intracranial pressure, thereby preventing neurodevelopmental complications. Despite the improved safety of craniosynostosis surgery, complications remain a concern [9]. 

A study conducted by Esparza et al. analyzed the complications associated with the surgical treatment of craniosynostosis and craniofacial syndromes involving 306 transcranial procedures [10]. The most frequent complication was postoperative hyperthermia, occurring in 13.17% of cases, followed by infections (8.10%), subcutaneous hematomas (6.08%), dural tears (5.06%), and cerebrospinal fluid leakage (2.7%). Notably, endoscopic-assisted osteotomies reported the lowest complication rate at 2.5%, while complete cranial vault remodeling had the highest rates of complications.

Despite these complications, all cases were resolved without permanent deficits, highlighting the effectiveness of surgical interventions [10]. Endoscopic correction offers certain advantages over open cranial vault reconstruction, including shorter surgery times, reduced blood loss, and quicker recovery periods, with fewer ICU and hospital stays. However, endoscopic techniques often require postoperative use of helmet-molding orthoses for several months to guide proper bone alignment and prevent suture refusion as the brain continues to grow. Both surgical approaches require long-term follow-up to monitor for late complications such as irregular bone contour, incomplete bone healing, or refusion of the sutures, which may necessitate reoperation. These considerations highlight the importance of tailored surgical planning and diligent postoperative care to optimize outcomes and minimize risks. However, even if the diagnosis is made at a later stage, the prognosis can be improved through intervention as seen in our case. The study by Gutierrez-Pineda F et al. demonstrated a 1% reoperation rate, a 95% success rate for achieving excellent aesthetic outcomes, and an 86% requirement for transfusions during procedures. Furthermore, the pooled complication rate was 2%, underscoring the minimal morbidity and highlighting the overall safety and efficacy of these surgical techniques [11]. Fortunately, our patient experienced no serious complications post-surgery. Regular follow-ups are essential to monitor head growth, detect increased ICP, and identify any potential issues.

## Conclusions

Oxycephaly poses a serious health concern that has the potential to affect both vision and neurological impairment. It is crucial for medical professionals to meticulously screen children for this condition. However, with the aid of surgical procedures, the prognosis for children with oxycephaly has significantly improved.
